# Protein expression profiling of plasma and lungs at different stages of metastatic development in a human triple negative breast cancer xenograft model

**DOI:** 10.1371/journal.pone.0215909

**Published:** 2019-05-01

**Authors:** Maria K. Tveitarås, Frode Selheim, Kristina Sortland, Rolf K. Reed, Linda Stuhr

**Affiliations:** 1 Department of Biomedicine, University of Bergen, Bergen, Norway; 2 Centre for Cancer Biomarkers, Norwegian Centre of Excellence, University of Bergen, Bergen, Norway; 3 Proteomic Unit (PROBE), Department of Biomedicine, University of Bergen, Bergen, Norway; University of South Alabama Mitchell Cancer Institute, UNITED STATES

## Abstract

The main objective of this study was to identify single proteins or protein networks that might be used as diagnostic biomarkers or for therapeutic purposes by evaluating the protein expression profiling of plasma and lungs at different stages of metastatic development in a human triple negative MDA-MB-231 breast cancer xenograft model. MDA-MB-231 tumour cells were injected into the mammary fat pads on one side of the groin area. The mice were sacrificed day 19 (pre-metastases) and day 54 (metastases). Non-injected mice served as controls. Plasma was collected and lungs harvested for both immunohistochemistry and protein analysis. The most striking observation in plasma was the initial reduction in haptoglobin level at the pre-metastatic stage, to a following significant increase in haptoglobin level at the metastatic stage, with a more than 4000-fold increase from the pre-metastatic to the metastatic phase. A corresponding increase in haptoglobin level was also found in lung tissue after metastasis. Fibrinogen beta chain also had a similar change in expression level in plasma as haptoglobin, however not as prominent. There were also changes in plasma thrombospondin-4 and transferrin receptor protein 1 levels, from an increase at the pre-metastatic stage, to a significant fall when metastases were established. This suggests that especially changes in haptoglobin, but also fibrinogen beta chain, thrombospondin-4 and transferrin receptor protein 1 is indicative of metastasis, at least in this breast cancer model, and should be further evaluated as general breast cancer biomarkers.

## Introduction

Breast cancer is the most common cancer among females worldwide. Each year, approximately 1.8 million patients are diagnosed with breast cancer, and more than 500 000 die from the disease [1]. Metastatic spread of breast cancer is responsible for 90% of breast cancer-related deaths and thus remains the most important negative prognostic predictor [1]. Therefore, recognition and understanding of the metastatic process is very important.

Metastasis, also classified as stage 4 breast cancers, is a complex process, which involves detachment of cancer cells from the primary tumour, extracellular matrix degradation, migration to, adhesion to and proliferation in the determined distant site. Research has identified the extracellular matrix (the “soil”) to play a crucial role for both primary tumour growth and metastasis. Recently the microenvironment was included in the “hallmarks” of cancer alongside those that are already well-established [2]. Thus, the development of metastases is dependent on the bidirectional communication between the cancer cell and the extracellular matrix [3]. Breast cancer spreads specifically to bone, lungs, liver, axillary glands and brain via the haematogenous route in humans. Because of this, we must assume that it may involve activation of host-specific signalling networks and cause changes in the plasma that arise after the primary tumour has developed. Triple negative breast cancers, such as the MDA-MB-231 used in our study, are particularly aggressive, are often associated with the BRCA1 mutation, and is commonly observed in younger women [4]. Once a triple negative breast cancer has become metastatic, the time from relapse of disease to death is a lot shorter than for other sub-types of breast cancer.

Our understanding of metastasis has increased through clinical studies [5]. However, sampling at late stages limits the ability to study the metastatic process over time. The aim of this project was to use the very aggressive and spontaneously metastasising triple negative cell line MDA-MB-231 *in vivo*, in an attempt to identify possible single proteins or proteome networks that might be used as diagnostic biomarkers or for therapeutic purposes.

## Material and methods

### Cell line

The highly metastatic human breast carcinoma cell line MDA-MB-231 was used. This cell line was obtained from the American type Culture Collection (Rockville, MS, USA). MDA-MB-231 is a triple-negative (lacking estrogen receptor, progesterone receptor and HER2 amplification) molecular subtype of breast cancer that was derived from pleural effusion of a breast cancer patient suffering from widespread metastasis years after removal of her primary tumour [6].

### Culturing cells

The MDA-MB-231 cells were cultured in Dulbecco’s Modified Eagle Medium (DMEM) (Bio-Whittaker, Verviers, Belgium) supplemented with 100 ml/L of foetal calf serum, 100 U/ml of penicillin and 100 μg/ml of streptomycin, 2% L-glutamine (Sigma-Aldrich, Steinheim, Germany). The cells were seeded into 75 cm^2^ plastic tissue culture flasks (NUNC, Roskilde, Denmark) and cultured as a monolayer in a humidified incubator set at 37° C with 5% CO_2_ and 95% air, until approximately 80% confluence.

### Animal model

A total of fifteen female NOD/SCID mice, weighing approximately 22-24g, were used in this study. The mice were all littermates and age matched. The animals were housed under diurnal light conditions and had free access to food and water *ad libitum*. The animal experiments were approved according to The Norwegian Food Safety Authority (Project number: 20157368). The Norwegian Food Safety Authority is the competent body responsible for authorising research projects involving animals in Norway. This is in accordance with the EU directive 2010/63, article 36. The staff members employed by The Norwegian Food Safety Authority are responsible veterinarians and biologist with special expertise regarding the use of research animals. University of Bergen has an Animal Welfare Body (equivalent to the IACUC) with special responsibility to provide advice on matters related to the welfare of animals, advise the staff on the application of the requirement of replacement, reduction and refinement, establish and review internal operational processes as regards monitoring, reporting and follow-up in relation to the welfare of animals and follow the development and outcome of projects, taking into account the effect on the animals used. The Animal Welfare Body review all protocols submitted for authorisation before they are sent to The Norwegian Food Safety Authority.

### Establishing primary tumours and metastasis

Cells were trypsinised and spun down for 4 min at 900 rpm, and further re-suspended in PBS. A total of 5 × 10^5^ MDA-MB-231 tumour cells in 0.15 mL PBS were injected orthotopically into the mammary fat pads on one side of the groin area. All animals were anesthetised using isoflurane (Rhone-Poulenc Chemicals, France) in combination with N_2_O and O_2_ during experiments. The animals were sacrificed during anaesthesia by cervical translocation. The experiments were either terminated on day 19 (pre-metastasis) or on day 54 (metastasis) post injection. Mice without tumours (non-injected) served as controls (n = 5 in each group).

### Blood samples

Blood was collected in sterilised Eppendorf tubes by heart puncture during deep terminal anaesthesia. Approximately 0.5–1 ml of blood was centrifuged at 3000 rpm for 8 min and plasma from each separate mouse was collected and stored at -80°C until analysed.

### Tissue preparation

After sacrifice, a suture was made on one bronchus. The other lung was fixed, using approximately 1 mL of Bouin’s solution (Gurr BDH Chemicals Ltd., Poole, UK) injected into the trachea. The lungs were then immediately dissected out. One lung was further fixed in new Bouin’s solution, washed in 70% ethanol, dehydrated and embedded in paraffin using standard procedures. Sections were stained with H & E staining and examined by light microscopy. From the other lung, 25 mg tissue was snap frozen and prepared for protein analysis.

In order to verify the presence of metastasis, 5 random lung sections from each animal were examined by microscopy (Nikon Digital Sight, Nikon Corporation).

### Protein analysis

#### Sample preparation for proteomics

Lung tissue samples (approximately 25 mg) were solubilised in 4% SDS / 0.1 M Tris-HCL with a blender before heating at 95°C for 5 min. Mouse plasma proteins were denatured in 4% SDS / 0.1 M Tris-HCL and heated at 95°C for 5 min. Samples were sonicated 5 cycles at 30% amplitude for 30 sec to shear nucleic acids. The cell debris was removed by centrifugation at 16000x g for 10 min. Protein concentrations in the lung and plasma supernatants were measured with the Pierce BCA Protein Assay Kit (Thermo Scientific). Further processing of each protein sample (30 μg) for LC-MS was done according to the filter-aided sample preparation (FASP) procedure [7], as recently described step by step elsewhere [8].

#### NanoLC mass spectrometry of tryptic peptides

Lyophilised Tryptic peptides (Centrivap with a Cold trap, Labconco, MO) were reconstituted in 1% aqueous formic acid (FA) / 2% acetonitrile (ACN), and 1 μg of each sample was automatically injected into an Ultimate 3000 RSLC system (Thermo Scientific, Sunnyvale, CA) connected online to a Q-Excative HF mass spectrometer (Thermo Scientific, Bremen, Germany). Peptides were pre-concentrated on a pre-column (Acclaim PepMap 100, 2cm x 75μm i.d. nanoViper column, packed with 3μm C18 beads), before separation of peptides on a 50 cm analytical column (Acclaim PepMap100 nanoViper column, 75 μm i.d. × 50 cm, packed with 3 μm C18 beads) at a flow rate of 250 nl/min. The peptides were separated utilising a biphasic gradient with 0.1% FA (solvent A) and 100% ACN (solvent B) for 120 min (5% B during trapping for 5 min followed by 5–7% B over 0.5 min, 7–22% B over 59.5 min, 22–35% B over 22 min, 35–90% over 5 min and then hold at 90% B for 10 min, then sloped to 5% B over 3 min and hold at 5% B for 15 min).

#### Q-excative HF

The mass spectrometer was operated in data-dependent-acquisition mode (DDA) to automatically switch between full scan MS and MS/MS acquisition. The scan range was 375–1500 m/z with resolution R = 120,000, and the automatic gain control (AGC) target was set to 3e6 with a maximum injection time (IT) of 100 ms. The MS/MS acquisition for the top 12 most intense peptides with intensity threshold >5e4, charge states 2 or more, were isolated to a target AGC value of 1e5 with IT 110 ms and resolution R = 60,000. The isolation window was 1.6 m/z, the isolation offset 1.3 m/z and the dynamic exclusion lasted for 20 seconds.

#### Label-free protein quantification and data analysis

The MaxQuant module (version 1.5.2.2) with the integrated MS1 peak intensity-based MaxLFQ software and Andromeda search engine were used for identification and label-free protein quantification of LC-MS data [9]. The raw MS data were searched against SwissProt *Mus musculus* database version 2016 01 (16747 entries)) with MaxQuant´s settings for label-free protein quantification as described previously [10, 11].

Briefly, Cysteine carbamidomethylation was set as fixed modification and protein N-terminal acetylation and oxidation of methionine as variable modifications. Two missed cleavages were allowed for digestion with trypsin. The label-free quantification (LFQ) mode [11] was selected with a minimal ratio set to two, and match-between-runs option permitted. Minimal peptide length was seven and the false discovery rate (FDR) was 0.01 for both peptides and proteins.

The proteomics raw files and data were uploaded to ProteomeXchange Consortium via PRIDE partner repository [12] with the dataset identifier PXD010248 and PXD010249.

### Western blot

Western blot was carried out on plasma samples directly. Protein concentration was measured using the Pierce BCA Protein Assay Kit (Life Technologies, Thermo Fisher Scientific, Waltham, MA, USA) according to the manufacturer’s specifications. Proteins were separated using one-dimensional SDS-PAGE. Protein samples were allowed to thaw on ice and further mixed with XT Sample Buffer, 4X (Bio-Rad Laboratories Inc, Hercules, CA, USA) in a 3:1 ratio. The proteins were denatured by boiling for 5 min at 90°C before loading onto a 12% Precise Protein Gel (Life Technologies, Thermo Fisher Scientific, Waltham, MA, USA) along with a SeeBlue Plus2 Prestained Standard (Invitrogen, Carlsbad, CA, USA) and an E-PAGE MagicMark Unstained Protein Standard (Life Technologies, Thermo Fisher Scientific, Waltham, MA, USA) marker as a reference for detecting proteins of interest. Electrophoresis was run for 10 min at 95 V to stack the proteins before running for 1 h at 110 V for separation. An Invitrogen iBlot Dry Blotting System (Life Technologies, Thermo Fisher Scientific, Waltham, MA, USA) was used to transfer the proteins from the gel onto a PDVF membrane. Ponceau S staining was used to determine equal loading of total protein (Sigma Aldrich, St. Louis, MO, USA), and washed with TBS-T until Ponceau S staining was removed. Membranes were incubated in I-Block (Life Technologies, Thermo Fisher Scientific, Waltham, MA, USA) for 1–2 h to saturate the membrane and prevent unspecific binding. The membrane was subsequently washed in TBS-T 3 x 10 min and incubated with the primary antibody at 4°C. On the following day, the membrane was washed in TBS-T and incubated with secondary antibody for 1.5 h at room temperature. After the final TBS-T wash, protein-bound antibodies were detected using the Pierce ECL Western Blotting Substrate (Life Technologies, Thermo Fisher Scientific, Waltham, MA, USA) and the Bio Rad Gel Doc XR (Bio-Rad Laboratories Inc, Hercules, CA, USA).

### Antibodies

Haptoglobin, ab231000, 1:1000, Abcam, Cambridge, UK. Goat polyclonal secondary antibody to Rabbit IgG (HRP) ab97051, 1:5000, Abcam, Cambridge, UK.

### Statistical and protein analysis tools

Analysis of significant protein fold changes were performed with paired *t*-tests and Z-statistics in Microsoft Excel [13]. Only proteins with p-value ≤ 0.05 for both Z- and *t*-tests were considered significant. Determination of significant enriched GO terms and associated proteins were done with the browser-based A Go Tool [14]. Proteins with significant fold changes (“foreground”) were compared against all identified and quantified proteins as background with keywords association set to GO Biological Process. The web-based STRING software (version 11.0) was used for detection of protein networks [15]. The Perseus (version 1.6.0.7) software was used for statistical multiple sample tests; MaxQuant raw data files were filtered, and reverse hits, potential contaminants and protein only identified by site were removed from the matrix. The data was log2-transformed and grouped into control, pre-metastasis and metastasis. Only proteins with at least 50% valid LFQ values in each group were used for statistical analysis. The multiple sample tests were set to Anova, S0 = 0, and with permutation based FDR used for truncation. FDR was set to 0.05, the number of randomisation to 250 and the q-values (adjusted p-values) were reported (equivalent to the corresponding FDR threshold). Quantified plasma and lung proteins (unique peptides ≥2) with both ANOVA p-values and FDR cutoff below 0.05 are marked (ANOVA significant) in S1 Table. Boxplot was created with SPSS (Statistic Package for the Social Science) statistic software (IBM SPSS Statistic for Widows, Version 25.0. Armonk, NY: IBM corp.).

## Results

In this study we investigated the proteomic profiles of lungs and plasma at different stages of breast cancer development in mice with MDA-MB-231 xenografts. The establishment of metastasis was confirmed histologically. Five plasma and 5 lung samples from each group (control, pre-metastatic and metastatic) were subjected to identical protein extraction and LC-MC and MS/MS procedures, however one control and two pre-metastatic plasma samples had to be discarded due to haemolysis of red blood cells (erythrocytes). We identified 389 proteins for the plasma proteome, of these 339 proteins had valid label-free quantification values after removal of potential contaminants and reverse hits.

Only proteins with p-value ≤ 0.05 for both Z- and *t*-tests were considered valid. Haptoglobin was the protein demonstrating greatest change by proteomics between groups and thus the most interesting. This was confirmed by western blot (see below).

### Metastases

**Fig 1:**
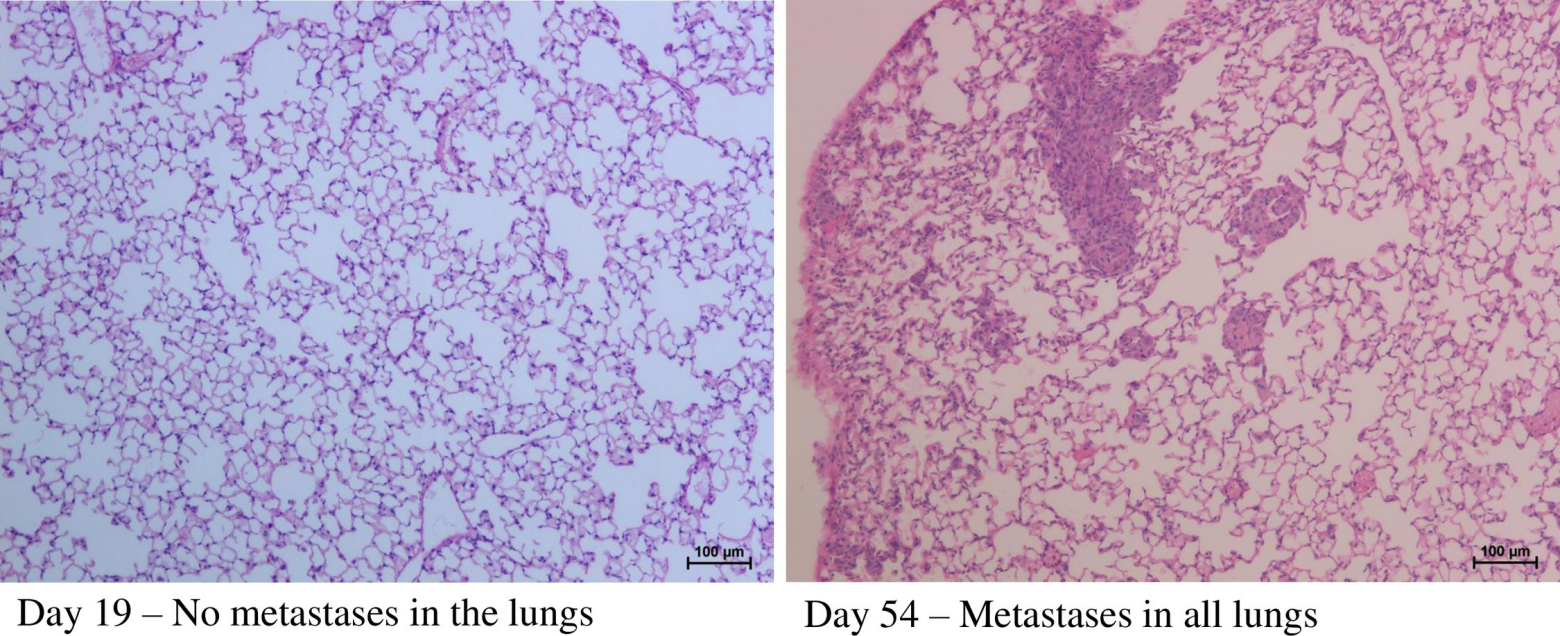
Lung morphology. 5 x10^5^ cells were injected in the mammary fat pad in the groin area. No metastases to the lungs were present after 19 days, however all animals had developed metastases by day 54. Scale bar: 100 μm.

Histological sections showed that none of the animals had developed lung metastases at day 19, however all animals had developed metastases at day 54 (Fig 1).

### Proteins in plasma

We identified 3013 proteins for the lung tissue proteome. Of these 2925 proteins had valid label-free quantification values after removal of potential contaminants and reverse hits.

**Fig 2:**
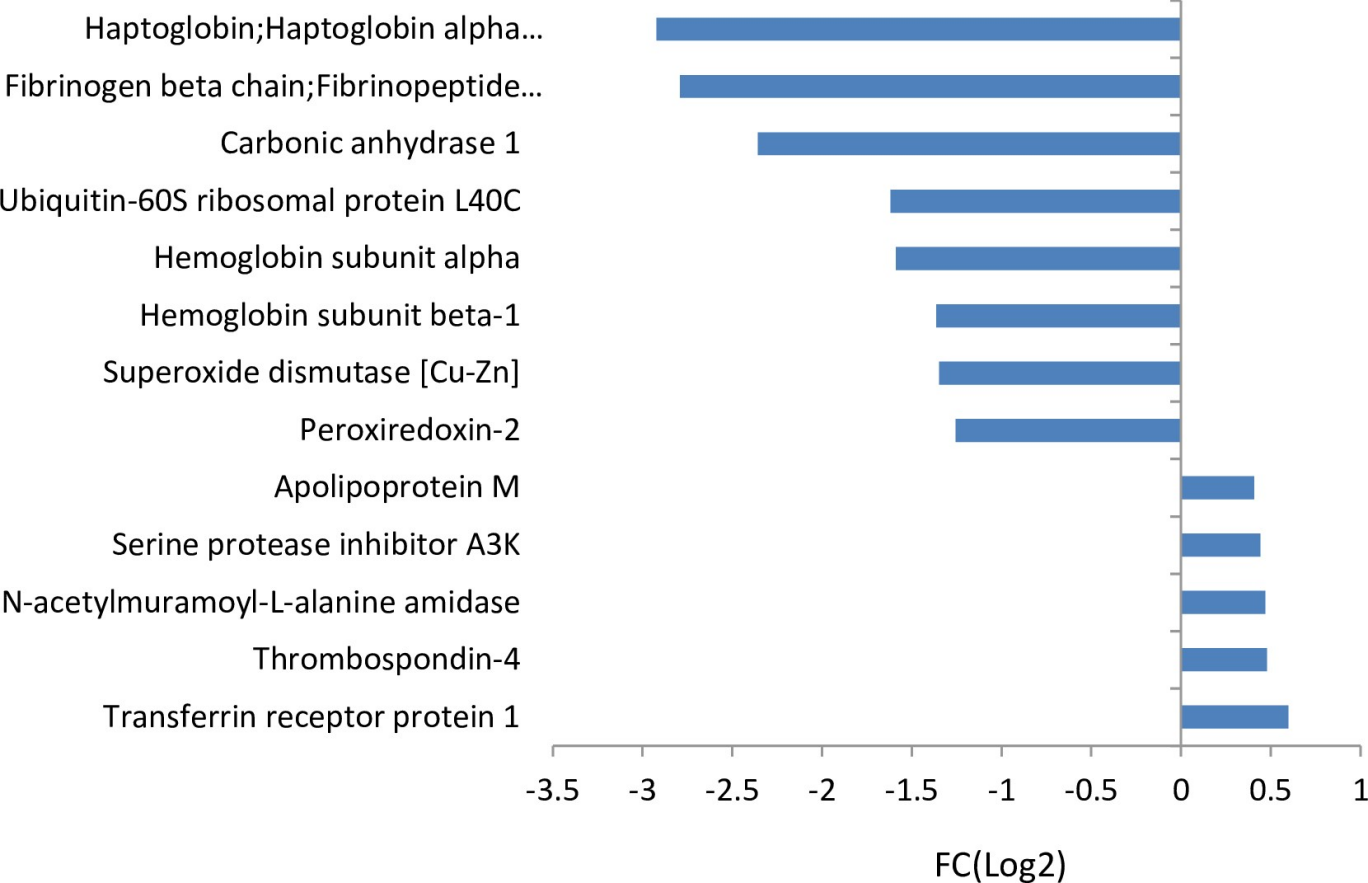
Protein fold changes (FC(Log2)) in plasma pre-metastasis (day 19) versus controls with p-value ≤ 0.05 for Z- and *t*-tests.

There were eight down-regulated and five up-regulated proteins in the pre-metastatic stage (pre-metastasis vs control) as shown in Fig 2, indicating changes in protein levels in plasma during early tumour progression. The eight significantly down-regulated proteins (FC(Log2)) > 1 and p-value ≤ 0.05 for Z- and *t*-tests) are correlated to oxygen stress, specifically by downregulation of antioxidants (peroxiredoxin-2, superoxide dismutase) and reduced oxygen transport (haptoglobin, haemoglobin subunit alpha and beta), while the 5 up-regulated proteins (p-value ≤ 0.05 for Z- and *t*-tests) display a variety of functions.

**Fig 3:**
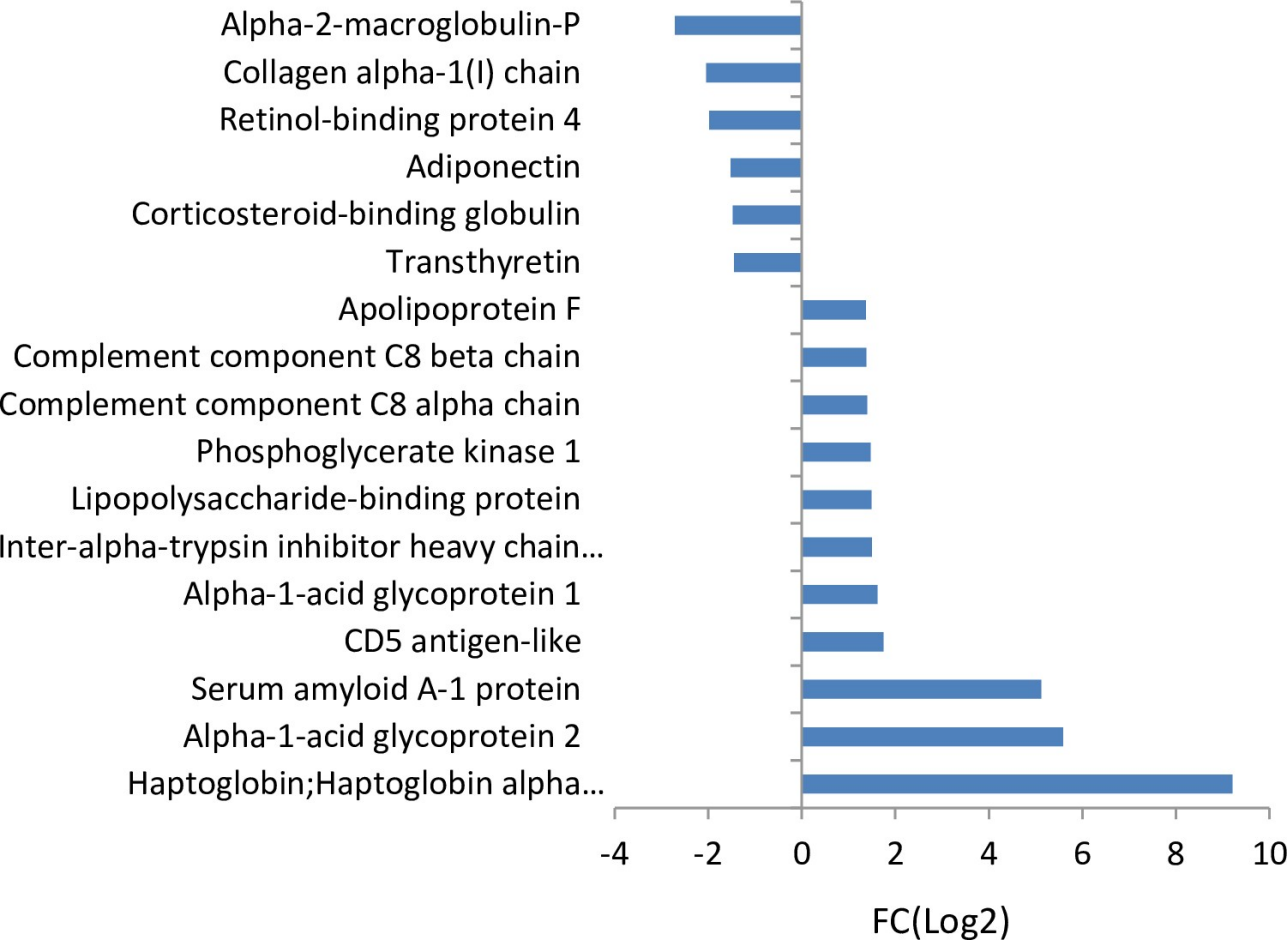
Protein fold changes (FC(Log2) ≥1 or ≤-1) in plasma, metastasis (day 54) versus controls with p-value ≤ 0.05 for Z- and *t*-tests.

**Fig 4:**
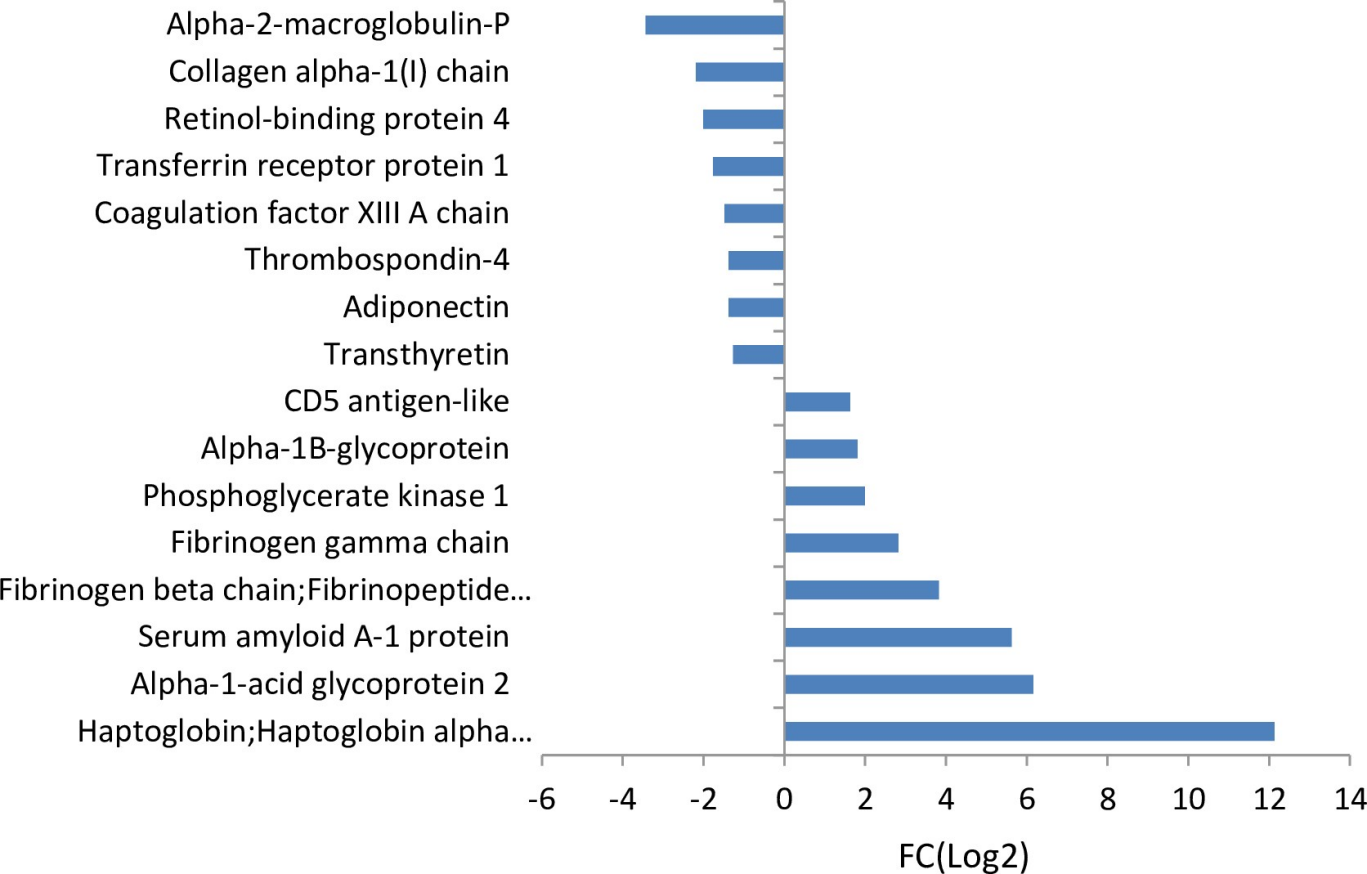
Protein fold changes (FC(Log2) ≥1 or ≤-1) in plasma, metastasis (day 54) versus pre-metastasis (day 19) with p-value ≤ 0.05 for Z- and *t*-tests.

There were six down-regulated proteins in the metastatic stage versus control plasma (metastasis vs control) and eight down-regulated proteins when the metastatic stage is compared to the pre-metastatic stage (metastasis vs pre-metastasis), as shown in Fig 3 and Fig 4 respectively. Five of these are identical in the two groups and have been identified to be involved in biological processes such as protein transport (corticosteroid-binding globulin, collagen type I alpha 1 chain, transthyretin, adiponectin, retinol binding protein 4) and hormone regulation (transthyretin, adiponectin, retinol-binding protein 4). Furthermore, 11 up-regulated proteins in the metastatic stage versus control plasma and eight proteins were up-regulated when comparing the metastatic to pre-metastatic stage, as shown in Fig 3 and Fig 4 respectively. Five of these are identical and they are all biologically connected to stress response (haptoglobin, alpha-1-acid glycoprotein 2, fibrinogen gamma chain, fibrinogen beta chain, serum amyloid A1).

### Metastasis signature in lung tissue

**Fig 5:**
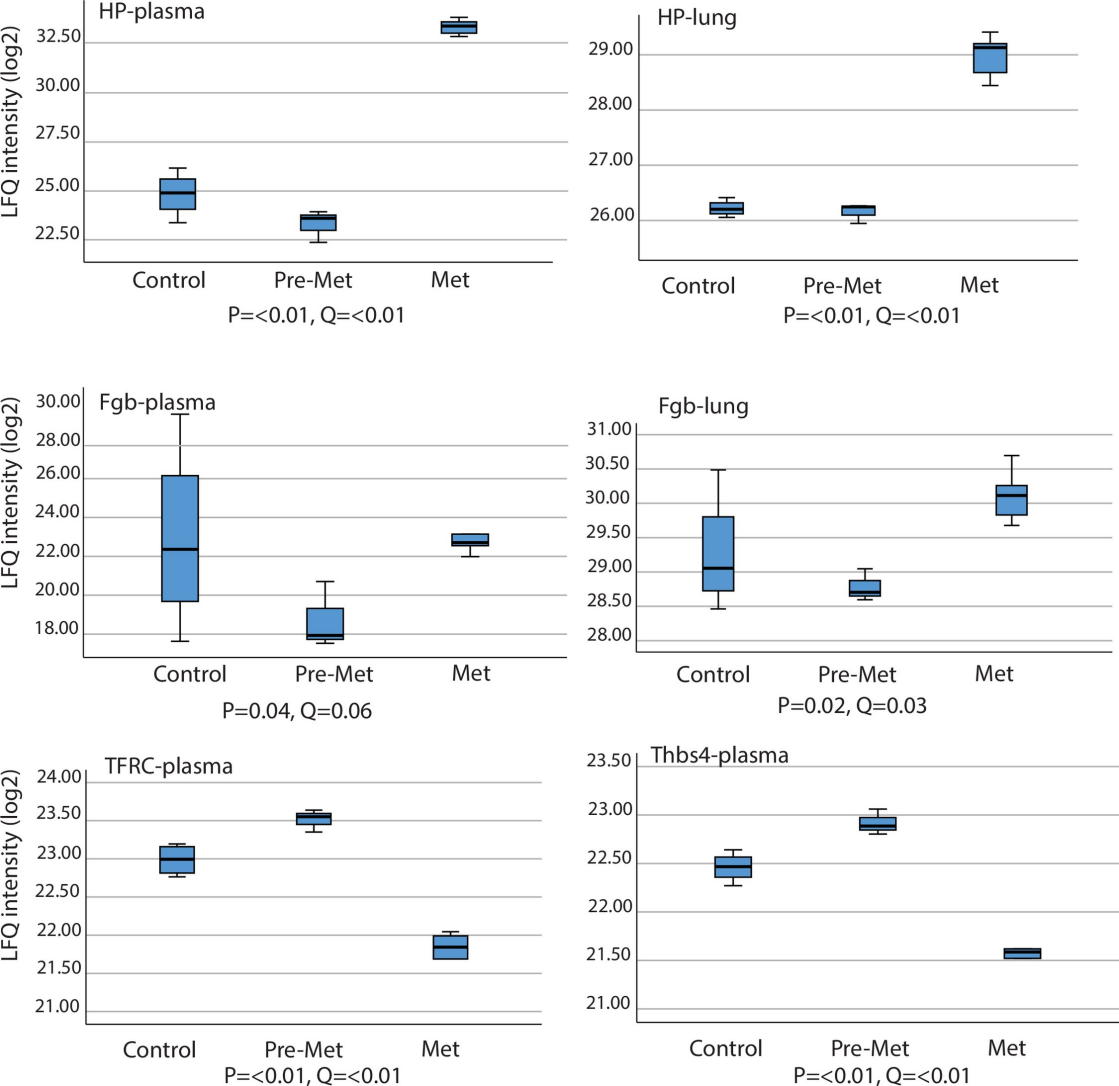
SPSS box plot to show the distribution (median, first quartile, third quartile, minimum and maximum value) of the LFQ intensity data for four potential biomarkers for metastatic breast cancer, including haptoglobin, fibrinogen beta chain, thrombospondin-4, and transferrin receptor protein.

71 proteins levels were significantly changed in lung tissue before metastasis had occurred (pre-metastasis vs control) (S2 Table). After metastases had been established in the lungs (metastasis vs pre-metastasis) we found that 288 proteins were enriched in lung tissue (S2 Table). The two most interesting proteins identified in lung tissue after metastasis were the ones that were also identifiable in plasma after metastasis; haptoglobin (FC(log2) 2.9) and fibrinogen beta chain (FC(log2) 1.4). To determine whether there is evidence for a true difference in expression between the different groups, a multiple sample test was conducted (S1 Table). An SPSS box plot to show the distribution of the LFQ intensity data for haptoglobin and fibrinogen beta chain are shown in Fig 5 for both lung tissue and plasma samples, as well as thrombospondin-4 and transferrin receptor protein for plasma.

**Fig 6:**
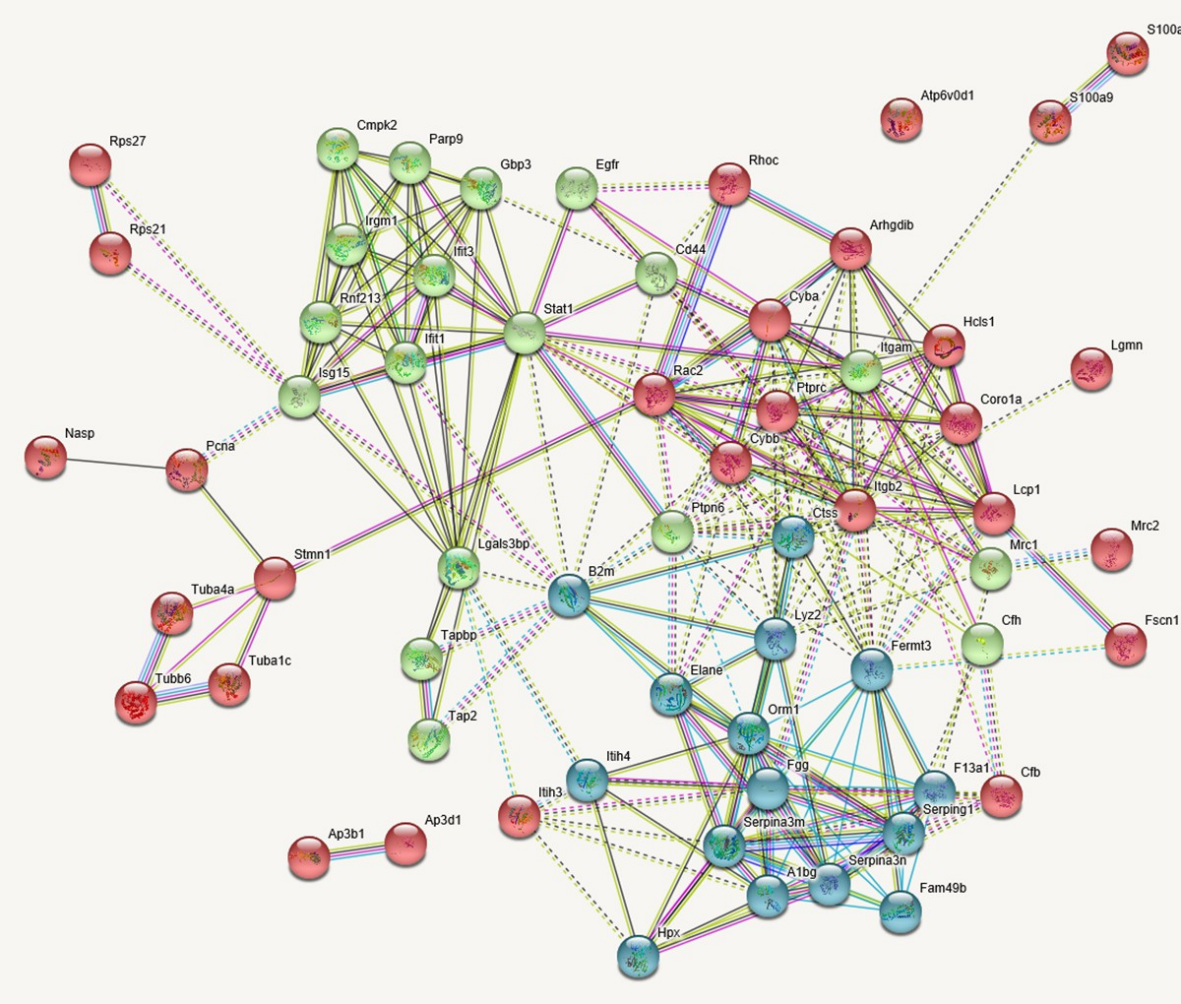
String analysis showed three dominating clusters when comparing metastasis (day 54) to the pre-metastatic group (day 19) in lung tissue. The blue cluster predominantly represented proteins involved with processes in inflammation, immune response, cell-cell interaction, haemostasis and haematopoiesis. The green cluster involved immune response, DNA damage, EGF receptor and haematopoiesis, and the red cluster involved metabolism, organisation of the cytoskeleton, cell movement and immune response.

Using the GO tool we found only one significant GO term with higher abundance in the pre-metastatic phase compared to the control group in lung tissue; mRNA-splicing via spliceosomes. More than thirty biological processes were identified as being up-regulated in lung tissue after metastasis compared to the pre-metastatic phase (S3 Table). A strict selection was made where the cut off was set at minimum 5 proteins in each enriched term, and p<0.05. After the selection, the remaining enriched terms were identified as being involved in processes such as cell adhesion, immune responses, positive regulation of superoxide anion generation and angiogenesis (S4 Table). Eleven enriched terms were identified as being higher in the pre-metastatic stage compared to after metastasis had occurred, when the same strict criteria as applied above there were nine remaining enriched terms. Those of interest in this study were the following; sarcomere organisation, positive regulation of peptidyl-tyrosine phosphorylation, response to hypoxia, protein localisation to plasma membrane, oxidation-reduction process and response to drug (S4 Table). Furthermore, string analysis showed three dominating clusters when comparing metastasis to the pre-metastatic group (Fig 6). One cluster predominantly represented proteins involved with immune responses, cell-cell interaction, haemostasis and haematopoiesis (blue cluster). The second cluster (green cluster) involved immune response, DNA damage, EGF receptor and haematopoiesis, and the third (red cluster) involved metabolism, cell movement and immune responses.

### Western blot

**Fig 7:**
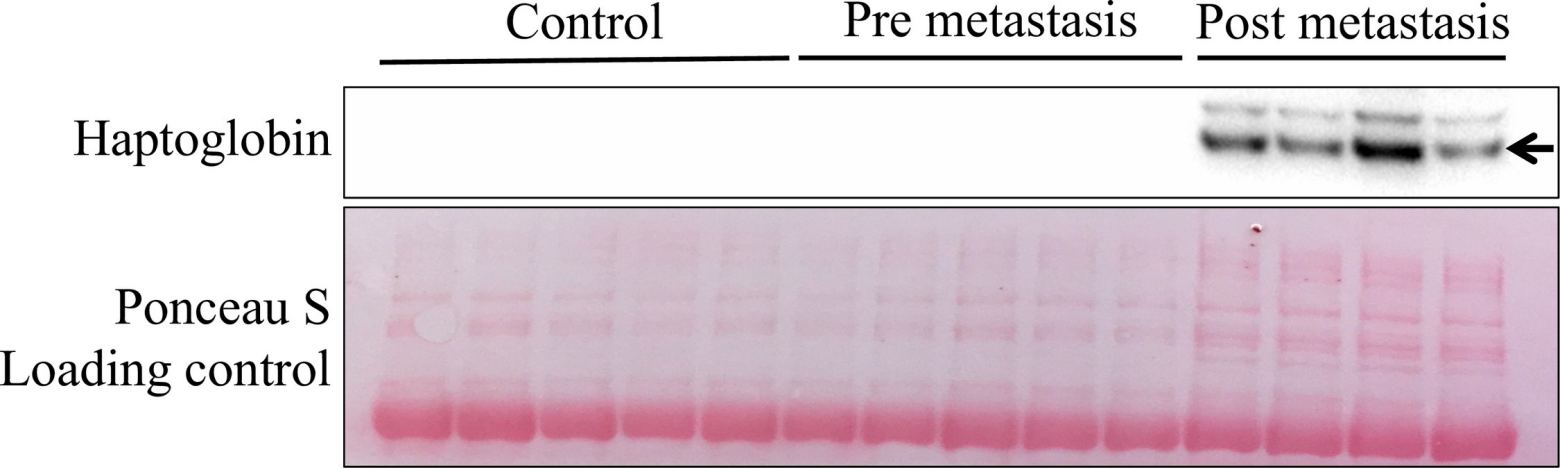
Western blot of plasma confirms a major up-regulation of haptoglobin after metastasis. Ponceau S was used as a loading control for total protein.

Western blot of the plasma samples showed a marked upregulation of haptoglobin in the post-metastatic state, confirming the proteomics results (Fig 7). Due to the dramatic increase of haptoglobin, with a more than 4000-fold up-regulation from pre-metastasis to the metastatic phase, imaging was not sensitive enough to enable visualisation of the bands in the control samples, because the metastatic samples were too dominant.

## Discussion

The present study identified a number of proteins and biological processes that showed altered levels in plasma and in lung tissue, both before and after spontaneous metastasis developed from primary breast cancer (MDA-MB-231) in the mammary fat pad in mice.

The most striking observation in plasma is that haptoglobin and fibrinogen beta chain seem to undergo a change in regulation at some point between the pre-metastatic phase (pre-metastatic vs control) and the metastatic phase (metastatic vs pre-metastatic), and this is particularly prominent for haptoglobin. Haptoglobin changes from a 3.25-fold (FC (Log2)) down-regulation at pre-metastasis, to a dramatic 12-fold (FC (Log2)) increase after metastasis, which is an actual 4096x upregulation. Haptoglobin binds free Hb released from erythrocytes and inhibits oxidative activity [16]. The initial drop in haptoglobin at the pre-metastatic phase could indicate haemolytic anaemia and most probably also be due to oxidative stress handling. In addition to acting as an antioxidant, other functions have been associated with an increase in haptoglobin levels. Haptoglobin has, for example, been found to be up-regulated in angiogenesis, it is an acute phase protein in inflammation, and an increase has also been observed in conjunction with transplants as a way of increasing inflammation in order to reject the new graft [17]. This situation could be related to the metastatic picture, as this also involves angiogenesis as well as an inflammatory response attempting to fight the metastatic spread. The great increase in haptoglobin after metastasis suggests that it could potentially serve as a biomarker for metastasis at least in our model. This corresponds to a case-control study investigating if it was possible to detect early stages of breast cancer by a serum panel of ten potential breast cancer markers, where haptoglobin was one of them [18]. Serum had been collected from healthy individuals, and was analysed at a later stage if they developed breast cancer. They were not able to detect any significant difference in the selected markers, however, they state that this could be due to the fact that blood samples were taken up to three years prior to the patient being diagnosed with breast cancer, and that it was therefore too early for detection of differences in levels of these proteins. They also stated that they did not know the sub-groups of breast cancer the patients had, which could affect the outcome. A different study looking at the expression of haptoglobin in patients with triple negative breast cancer identified it to be a potential biomarker [19]. These workers identified a correlation between expression levels of haptoglobin and patient outcome, where the patients with the highest levels had a poorer prognosis. They therefore suggested haptoglobin to be both a prognostic marker, as well as a potential therapeutic target. Since this is in line with our results, it is thus tempting to suggest that haptoglobin is a possible specific biomarker for triple negative breast cancer. Haptoglobin has also been indicated as a serum biomarker for ovarian cancer [20, 21] and small cell lung cancer [22]. One study has also reported that haptoglobin could participate with STAT3 and HIF-1a in promoting angiogenesis and cell migration, and thus contributes to driving tumour growth and invasion [23]. In addition, our results show that the upregulation of haptoglobin is also reflected in the lungs after the establishment of metastasis, where we identified an almost 3-fold upregulation of the protein.

In the present study fibrinogen beta chain was shown to be down-regulated around 3-fold (FC(Log2)) in the pre-metastatic phase, however after metastasis it is up-regulated about 4-fold (FC (Log2)). Fibrinogen (alpha, beta and gamma chain) has been found to be up-regulated in advanced breast cancer [24]. It also plays an important role in cancer pathophysiology, and it is a determinant of metastatic potential as fibrinogen deficiency diminished the formation of lung metastasis in a murine melanoma and a murine lung carcinoma model. However, it does not seem to play a role in the growth of established metastases [25]. In the present study the initial pre-metastatic reduction, and subsequent increase after metastasis in both haptoglobin and fibrinogen beta chain found in the present study shows that these proteins might be good biomarkers in the prediction of metastasis.

Thrombospondin-4 and transferrin receptor protein 1 also exhibited similar changes in expression levels, however these were not as prominent as the changes in levels seen in haptoglobin and fibrinogen beta chain. Thrombospondin-4 and transferrin receptor protein 1 had an approximate fold change (FC(log2)) upregulation of 0.5 and 0.6 respectively before the establishment of metastasis (B vs A). After metastasis thrombospondin-4 had a down-regulated fold change of approximately -2 (FC (Log4)), whilst transferrin receptor protein 1 had a fold change (FC (Log2)) of -2.5. Thrombospondin 4 is up-regulated at the pre-metastatic stage. It is an adhesive glycoprotein and is said to be activated during tissue remodelling processes such as wound healing and cancer [26]. Thrombospondin 4 contributes to the activation of stromal responses exhibited during tumour progression and this may facilitate invasion of tumour cells in breast cancer [27]. The up-regulation of this protein is therefore indicative of enhanced cellular migration and proliferation, and thus a possible biomarker for metastatic potential.

The eight down-regulated proteins in the pre-metastasis stage (pre-metastatic vs control) are correlated to oxygen stress, while the 5 up-regulated ones have varying functions. Two anti-oxidant enzymes were significantly down-regulated. The superoxide dismutase and peroxiredoxin-2 are both antioxidants, and thus reduces the damage induced by free radicals in the body. Hence, a reduced anti-oxidant level is indicative of enhanced free radical levels in this first step towards metastasis. Furthermore, there is downregulation of haemoglobin (both subunits), which is a protein that is involved in O_2_ transport, and an up-regulation of transferrin receptor protein, which is a protein responsible for iron transport into cells. Both of these indicate anaemia/reduced O_2_ transport levels prior to the development of metastases. An increase in transferrin receptor protein more specifically indicates iron-deficiency anaemia [28]. Anaemia is known to be associated with poor outcome in patients with breast cancer [29, 30]. Apolipoprotein M, which is also up-regulated, is involved in lipid transport but its biological function remains to be elucidated [31]. Apolipoprotein M mRNA has however been closely associated with nodal metastasis in colon cancer [32].

No changes in biological processes of interest were observed in the lung tissue before metastases were established when comparing these to the controls (pre-metastatic vs control). However, after the development of metastases many biological processes were altered (metastatic vs pre-metastatic). At the pre-metastatic stage processes involved in drug response, oxidation-reduction processes, response to hypoxia and protein localisation to plasma membrane were more active than after development of metastases, and are therefore of interest. Hypoxia is known to be a prognostic marker for poor patient outcome [33, 34], and previous studies from our group have demonstrated that the use of hyperbaric oxygen treatment for the purpose of reducing hypoxia limits tumour growth and development of metastasis [35–41]. An up-regulation of hypoxic response at this point could therefore suggest this to be a crucial stage for the development of metastasis. After the establishment of metastasis up-regulated biological processes of interest were related to immune responses and cell-matrix adhesion where fibrinogen beta chain contributes to both these processes, and positive regulation of angiogenesis. These processes suggest accelerated tumour growth, establishment of metastases, and that the tumour has ability to promote angiogenesis for the purpose of increasing supply of nutrients. The protein we discovered to be most up-regulated after metastasis, haptoglobin, was found to contribute to the biological process of immunity, more specifically defence against bacteria.

String analysis showed three dominating clusters when comparing the metastatic to the pre-metastatic stage which all showed general interactions between processes involved in immune response, cell-cell interaction, cell movement, DNA-damage, haemostasis and haematopoesis. These are all expected processes in tumour development and the establishment of metastasis.

## Conclusion

The procedure of collecting a blood sample from a patient is a simple procedure, and a plasma sample is thus easily obtainable from patients. It is therefore highly favourable to be able to identify proteins in plasma that could aid the identification of breast cancer at an early stage. In plasma the most prominent changes in protein expression were haptoglobin and fibrinogen beta chain, from a marked down-regulation before development of metastasis to significant up-regulation after the establishment of metastasis, with haptoglobin being the most striking observation. This is also reflected in lung tissue after metastasis. There were also changes in plasma thrombospondin-4 and transferring receptor protein 1 levels, from an increase at the pre-metastatic stage, to a significant fall when metastases were established. Taken together this suggests that especially changes in haptoglobin levels, but also fibrinogen beta chain, thrombospondin-4 and transferrin receptor protein 1 levels are indicative of metastasis in at least this breast cancer model, and should be further evaluated as general breast cancer biomarkers.

## Supporting information

S1 Table Multiple sample tests set to Anova, S0 = 0, and with permutation based FDR used for truncation. FDR was set to 0.05, the number of randomisation to 250 and the q-values (adjusted p-values) were reported (equivalent to the corresponding FDR threshold). Quantified plasma and lung proteins (unique peptides ≥2) with both ANOVA p-values and FDR cutoff below 0.05 are marked (ANOVA significant)
(XLSX)

S2 Table Protein fold changes (FC(Log2)) in lung tissue showing pre-metastasis versus control (B v A), and metastasis versus pre-metastasis (C v B). P-value ≤ 0.05 for Z- and *t*-tests.
(XLSX)

S3 Table All statistically significant biological processes identified in lung tissue when comparing the pre-metastatic to the control group. A strict selection was made where the cut off was set to a minimum of 5 proteins in each enriched term, with FC >2 and p<0.05.
(XLS)

S4 Table All statistically significant biological processes identified in lung tissue when comparing the metastatic to the pre-metastatic group. A strict selection was made where the cut off was set to a minimum of 5 proteins in each enriched term, with FC >2 and p<0.05.
(XLS)

## References

[1] Global Burden of Disease Cancer C, Fitzmaurice C, Dicker D, Pain A, Hamavid H, Moradi-Lakeh M, et al The Global Burden of Cancer 2013. JAMA oncology. 2015;1(4):505–27. 10.1001/jamaoncol.2015.0735 26181261 PMC4500822

[2] Hanahan D, Coussens LM. Accessories to the crime: functions of cells recruited to the tumor microenvironment. Cancer cell. 2012;21(3):309–22. 10.1016/j.ccr.2012.02.022 . 22439926

[3] Joyce JA, Pollard JW. Microenvironmental regulation of metastasis. Nature reviews Cancer. 2009;9(4):239–52. 10.1038/nrc2618 19279573 PMC3251309

[4] Hudis CA, Gianni L. Triple-negative breast cancer: an unmet medical need. The oncologist. 2011;16 Suppl 1:1–11. 10.1634/theoncologist.2011-S1-01 . 21278435

[5] Kimbung S, Loman N, Hedenfalk I. Clinical and molecular complexity of breast cancer metastases. Seminars in cancer biology. 2015;35:85–95. 10.1016/j.semcancer.2015.08.009 . 26319607

[6] Cailleau R, Olive M, Cruciger QV. Long-term human breast carcinoma cell lines of metastatic origin: preliminary characterization. In vitro. 1978;14(11):911–5. . 730202 10.1007/BF02616120

[7] Wisniewski JR, Zougman A, Nagaraj N, Mann M. Universal sample preparation method for proteome analysis. Nature methods. 2009;6(5):359–62. 10.1038/nmeth.1322 . 19377485

[8] Hernandez-Valladares M, Aasebo E, Mjaavatten O, Vaudel M, Bruserud O, Berven F, et al Reliable FASP-based procedures for optimal quantitative proteomic and phosphoproteomic analysis on samples from acute myeloid leukemia patients. Biological procedures online. 2016;18:13 10.1186/s12575-016-0043-0 27330413 PMC4915068

[9] Cox J, Neuhauser N, Michalski A, Scheltema RA, Olsen JV, Mann M. Andromeda: a peptide search engine integrated into the MaxQuant environment. Journal of proteome research. 2011;10(4):1794–805. 10.1021/pr101065j . 21254760

[10] Aasebo E, Mjaavatten O, Vaudel M, Farag Y, Selheim F, Berven F, et al Freezing effects on the acute myeloid leukemia cell proteome and phosphoproteome revealed using optimal quantitative workflows. Journal of proteomics. 2016;145:214–25. 10.1016/j.jprot.2016.03.049 . 27107777

[11] Cox J, Hein MY, Luber CA, Paron I, Nagaraj N, Mann M. Accurate proteome-wide label-free quantification by delayed normalization and maximal peptide ratio extraction, termed MaxLFQ. Molecular & cellular proteomics: MCP. 2014;13(9):2513–26. 10.1074/mcp.M113.031591 24942700 PMC4159666

[12] Vizcaino JA, Deutsch EW, Wang R, Csordas A, Reisinger F, Rios D, et al ProteomeXchange provides globally coordinated proteomics data submission and dissemination. Nature biotechnology. 2014;32(3):223–6. 10.1038/nbt.2839 24727771 PMC3986813

[13] Arntzen MO, Koehler CJ, Barsnes H, Berven FS, Treumann A, Thiede B. IsobariQ: software for isobaric quantitative proteomics using IPTL, iTRAQ, and TMT. Journal of proteome research. 2011;10(2):913–20. 10.1021/pr1009977 . 21067241

[14] Scholz C, Lyon D, Refsgaard JC, Jensen LJ, Choudhary C, Weinert BT. Avoiding abundance bias in the functional annotation of post-translationally modified proteins. Nature methods. 2015;12(11):1003–4. 10.1038/nmeth.3621 . 26513550

[15] Szklarczyk D, Morris JH, Cook H, Kuhn M, Wyder S, Simonovic M, et al The STRING database in 2017: quality-controlled protein-protein association networks, made broadly accessible. Nucleic acids research. 2017;45(D1):D362–D8. 10.1093/nar/gkw937 27924014 PMC5210637

[16] Chang YK, Lai YH, Chu Y, Lee MC, Huang CY, Wu S. Haptoglobin is a serological biomarker for adenocarcinoma lung cancer by using the ProteomeLab PF2D combined with mass spectrometry. American journal of cancer research. 2016;6(8):1828–36. 27648369 PMC5004083

[17] Maffei M, Barone I, Scabia G, Santini F. The Multifaceted Haptoglobin in the Context of Adipose Tissue and Metabolism. Endocrine reviews. 2016;37(4):403–16. 10.1210/er.2016-1009 . 27337111

[18] Opstal-van Winden AW, Rodenburg W, Pennings JL, van Oostrom CT, Beijnen JH, Peeters PH, et al A bead-based multiplexed immunoassay to evaluate breast cancer biomarkers for early detection in pre-diagnostic serum. International journal of molecular sciences. 2012;13(10):13587–604. 10.3390/ijms131013587 23202969 PMC3497343

[19] Tabassum U, Reddy O, Mukherjee G. Elevated serum haptoglobin is associated with clinical outcome in triple-negative breast cancer patients. Asian Pacific journal of cancer prevention: APJCP. 2012;13(9):4541–4. . 23167376 10.7314/apjcp.2012.13.9.4541

[20] Ahmed N, Barker G, Oliva KT, Hoffmann P, Riley C, Reeve S, et al Proteomic-based identification of haptoglobin-1 precursor as a novel circulating biomarker of ovarian cancer. British journal of cancer. 2004;91(1):129–40. 10.1038/sj.bjc.6601882 15199385 PMC2364749

[21] Ye B, Cramer DW, Skates SJ, Gygi SP, Pratomo V, Fu L, et al Haptoglobin-alpha subunit as potential serum biomarker in ovarian cancer: identification and characterization using proteomic profiling and mass spectrometry. Clinical cancer research: an official journal of the American Association for Cancer Research. 2003;9(8):2904–11. . 12912935

[22] Bharti A, Ma PC, Maulik G, Singh R, Khan E, Skarin AT, et al Haptoglobin alpha-subunit and hepatocyte growth factor can potentially serve as serum tumor biomarkers in small cell lung cancer. Anticancer research. 2004;24(2C):1031–8. . 15154618

[23] Oh MK, Park HJ, Kim NH, Park SJ, Park IY, Kim IS. Hypoxia-inducible factor-1alpha enhances haptoglobin gene expression by improving binding of STAT3 to the promoter. The Journal of biological chemistry. 2011;286(11):8857–65. 10.1074/jbc.M110.150557 21224490 PMC3058976

[24] Dowling P, Palmerini V, Henry M, Meleady P, Lynch V, Ballot J, et al Transferrin-bound proteins as potential biomarkers for advanced breast cancer patients. BBA clinical. 2014;2:24–30. 10.1016/j.bbacli.2014.08.004 26673961 PMC4633920

[25] Palumbo JS, Kombrinck KW, Drew AF, Grimes TS, Kiser JH, Degen JL, et al Fibrinogen is an important determinant of the metastatic potential of circulating tumor cells. Blood. 2000;96(10):3302–9. . 11071621

[26] Kazerounian S, Yee KO, Lawler J. Thrombospondins in cancer. Cellular and molecular life sciences: CMLS. 2008;65(5):700–12. 10.1007/s00018-007-7486-z 18193162 PMC2752021

[27] McCart Reed AE, Song S, Kutasovic JR, Reid LE, Valle JM, Vargas AC, et al Thrombospondin-4 expression is activated during the stromal response to invasive breast cancer. Virchows Archiv: an international journal of pathology. 2013;463(4):535–45. 10.1007/s00428-013-1468-3 . 23942617

[28] Skikne BS. Serum transferrin receptor. American journal of hematology. 2008;83(11):872–5. 10.1002/ajh.21279 . 18821709

[29] Bottini A, Berruti A, Brizzi MP, Bersiga A, Generali D, Allevi G, et al Pretreatment haemoglobin levels significantly predict the tumour response to primary chemotherapy in human breast cancer. British journal of cancer. 2003;89(6):977–82. 10.1038/sj.bjc.6601216 12966412 PMC2376950

[30] Henke M, Laszig R, Rube C, Schafer U, Haase KD, Schilcher B, et al Erythropoietin to treat head and neck cancer patients with anaemia undergoing radiotherapy: randomised, double-blind, placebo-controlled trial. Lancet. 2003;362(9392):1255–60. 10.1016/S0140-6736(03)14567-9 . 14575968

[31] Huang LZ, Gao JL, Pu C, Zhang PH, Wang LZ, Feng G, et al Apolipoprotein M: Research progress, regulation and metabolic functions (Review). Molecular medicine reports. 2015;12(2):1617–24. 10.3892/mmr.2015.3658 . 25901639

[32] Mu QF, Luo GH, Chen LJ, Wei J, Zheng L, Zhang XY, et al [Apolipoprotein M expression in human colorectal cancer tissues and its clinicopathological relevance]. Zhonghua wei chang wai ke za zhi = Chinese journal of gastrointestinal surgery. 2012;15(8):855–8. . 22941695

[33] Harris AL. Hypoxia—a key regulatory factor in tumour growth. Nature reviews Cancer. 2002;2(1):38–47. 10.1038/nrc704 . 11902584

[34] Vaupel P, Harrison L. Tumor hypoxia: causative factors, compensatory mechanisms, and cellular response. The oncologist. 2004;9 Suppl 5:4–9. 10.1634/theoncologist.9-90005-4 . 15591417

[35] Moen I, Jevne C, Wang J, Kalland KH, Chekenya M, Akslen LA, et al Gene expression in tumor cells and stroma in dsRed 4T1 tumors in eGFP-expressing mice with and without enhanced oxygenation. BMC cancer. 2012;12:21 10.1186/1471-2407-12-21 22251838 PMC3274430

[36] Moen I, Oyan AM, Kalland KH, Tronstad KJ, Akslen LA, Chekenya M, et al Hyperoxic treatment induces mesenchymal-to-epithelial transition in a rat adenocarcinoma model. PloS one. 2009;4(7):e6381 10.1371/journal.pone.0006381 19636430 PMC2712688

[37] Moen I, Stuhr LE. Hyperbaric oxygen therapy and cancer—a review. Targeted oncology. 2012;7(4):233–42. 10.1007/s11523-012-0233-x 23054400 PMC3510426

[38] Moen I, Tronstad KJ, Kolmannskog O, Salvesen GS, Reed RK, Stuhr LE. Hyperoxia increases the uptake of 5-fluorouracil in mammary tumors independently of changes in interstitial fluid pressure and tumor stroma. BMC cancer. 2009;9:446 10.1186/1471-2407-9-446 20017908 PMC2805681

[39] Raa A, Stansberg C, Steen VM, Bjerkvig R, Reed RK, Stuhr LE. Hyperoxia retards growth and induces apoptosis and loss of glands and blood vessels in DMBA-induced rat mammary tumors. BMC cancer. 2007;7:23 10.1186/1471-2407-7-23 17263869 PMC1797183

[40] Stuhr LE, Iversen VV, Straume O, Maehle BO, Reed RK. Hyperbaric oxygen alone or combined with 5-FU attenuates growth of DMBA-induced rat mammary tumors. Cancer letters. 2004;210(1):35–40. 10.1016/j.canlet.2004.02.012 . 15172118

[41] Yttersian Sletta K, Tveitaras MK, Lu N, Engelsen AST, Reed RK, Garmann-Johnsen A, et al Oxygen-dependent regulation of tumor growth and metastasis in human breast cancer xenografts. PloS one. 2017;12(8):e0183254 10.1371/journal.pone.0183254 28832662 PMC5568407

